# Adipose tissue morphology, imaging and metabolomics predicting cardiometabolic risk and family history of type 2 diabetes in non-obese men

**DOI:** 10.1038/s41598-020-66199-z

**Published:** 2020-06-19

**Authors:** Aidin Rawshani, Björn Eliasson, Araz Rawshani, Josefin Henninger, Adil Mardinoglu, Åsa Carlsson, Maja Sohlin, Maria Ljungberg, Ann Hammarstedt, Annika Rosengren, Ulf Smith

**Affiliations:** 1 The Lundberg Laboratory for Diabetes Research, Department of Molecular and Clinical Medicine, the Sahlgrenska Academy at the University of Gothenburg, Gothenburg, Sweden; 2 Science for Life Laboratory, KTH - Royal Institute of Technology, Stockholm, SE-17121 Sweden; 3 Centre for Host-Microbiome Interactions, Faculty of Dentistry, Oral & Craniofacial Sciences, King’s College London, London, SE1 9RT United Kingdom; 4 Institute of Clinical Sciences, Department of Radiation Physics, Sahlgrenska Academy at the University of Gothenburg, Gothenburg, Sweden

**Keywords:** Predictive markers, Type 2 diabetes, Obesity

## Abstract

We evaluated the importance of body composition, amount of subcutaneous and visceral fat, liver and heart ectopic fat, adipose tissue distribution and cell size as predictors of cardio-metabolic risk in 53 non-obese male individuals. Known family history of type 2 diabetes was identified in 25 individuals. The participants also underwent extensive phenotyping together with measuring different biomarkers and non-targeted serum metabolomics. We used ensemble learning and other machine learning approaches to identify predictors with considerable relative importance and their intricate interactions. Visceral fat and age were strong individual predictors of ectopic fat accumulation in liver and heart along with markers of lipid oxidation and reduced glucose tolerance. Subcutaneous adipose cell size was the strongest individual predictor of whole-body insulin sensitivity and also a marker of visceral and ectopic fat accumulation. The metabolite 3-MOB along with related branched-chain amino acids demonstrated strong predictability for family history of type 2 diabetes.

## Introduction

Overweight, obesity and a sedentary life style are major causes of the current global Type 2 diabetes epidemic. Excess body weight promotes insulin resistance and the expanded adipose tissue plays a critical role for this. The subcutaneous adipose tissue is the largest and preferred site to accumulate excess fat but it is limited in its ability to recruit new cells in adults and, thus, cell expansion is the major way of accommodating excess lipids^1,2^. Expanded subcutaneous adipose cells tend to become proinflammatory, insulin resistant and to have increased lipolysis. In addition, visceral fat is increasingly used for lipid storage while ectopic fat accumulates in the liver, heart and other tissues, which also enhances insulin resistance and the dysmetabolic state^2,3^.

Ability to differentiate new subcutaneous adipose cells is reduced in adults with expanded cells, not because of reduced number of precursor cells but as a consequence of increased cell senescence^4^. Importantly, individuals with type 2 diabetes (T2D)^5^, and also non-diabetic individuals with family history for T2D (First-Degree Relatives), have inappropriately expanded adipose cells in relation to their BMI^6,7^ due to the reduced subcutaneous adipogenesis in the presence of increased senescent progenitor cells^4^. Conceptually, there should be a close interaction between ability to store excess lipids in subcutaneous fat and the accumulation of visceral fat, ectopic fat and development of a dysmetabolic state. The associations and potential regulation exercised by subcutaneous adipose tissue lipid accumulation and cellular morphology on overall metabolic risk profile with imaging and metabolomics data and relation to family history have, to our knowledge, never been examined before in man.

Several large clinical studies have shown that individuals with known genetic markers of insulin resistance are characterized by reduced subcutaneous adipose tissue^8,9^, which is consistent with our findings of reduced adipogenesis in first-degree relatives and T2D^4,10^. A further level of complexity is that there is considerable heterogeneity among different adipose tissue depots in regards to their metabolic regulation and endocrine secretion.

In the present study, we sought to identify novel predictors and pathways that can improve the characterization of patho-physiological alterations associated with metabolic risk. Extensive identification of biomarkers and their intricate interactions should allow new insight into the patho-physiological progression and risk of developing T2D and its consequences.

For this purpose, we used unbiased machine learning approaches to analyze this comprehensive database consisting of extensive phenotyping combined with radiological imaging and body composition analysis, subcutaneous adipose tissue mass and morphology, and non-targeted serum metabolomics in healthy lean/overweight individuals. Approximately half of our cohort consisted of individuals with a family history of T2D (FDR) and these were compared to subjects without known heredity.

## Results

### Study population

We included 53 men; 25 with a known family history of T2D (First-Degree Relatives; FDR) and 28 without. Mean age was 42 ± 8 years but FDR were significantly younger (39.3 vs 45.6 yrs, p < 0.004). The groups had similar body mass index and other basal phenotypic data with only minor differences as demonstrated in Table 1. Thus, the cohort exhibited no metabolic abnormalities and was comprised of middle-aged, healthy, lean or mildly overweight non-diabetic individuals with normal liver function and blood pressure levels and without any ongoing pharmacological therapy. The complete variable list of baseline characteristics for predictors used in the statistical models are presented in Supplementary Table 1.

**Table 1:** Baseline characteristics for clinical variables and radiological examinations in first-degree relatives and control subjects without diabetes.

Characteristics	Control subjects	First-degree relatives	P-value	Overall
Number	28	25		53
Age – yr	39.29 (7.95)*	45.60 (7.34)	0.004	42.26 (8.23)
**Blood pressure – mm hg**				
Diastolic blood pressure	79.54 (9.34)	82.20 (10.70)	0.338	80.79 (9.99)
Systolic blood pressure	125.86 (12.51)	128.92 (11.98)	0.368	127.30 (12.24)
Body mass index, kg/m^2^	25.26 (3.88)	25.93 (3.12)	0.492	25.57 (3.52)
Glycated hemoglobin (mmol/mol)†	32.50 (2.42)	33.31 (2.16)	0.207	32.88 (2.31)
Waist to height (mean)	88.77 (10.89)	92.00 (7.83)	0.226	90.29 (9.62)
Serum creatinine, μmol/L	90.46 (11.37)	86.12 (9.09)	0.134	88.42 (10.49)
Waist to hip ratio	0.87 (0.06)	0.90 (0.05)	0.090	0.89 (0.06)
**Oral glucose tolerance test (OGTT)**				
Fasting plasma glucose, mmol/L	4.86 (0.43)	5.01 (0.39)	0.211	4.93 (0.41)
Fasting serum insulin, pmol/L	47.82 (25.49)	42.64 (18.11)	0.403	45.38 (22.26)
Plasma glucose levels after 60 min	7.71 (2.32)	7.72 (1.87)	0.983	7.71 (2.10)
Plasma glucose levels after 2h	5.12 (1.82)	5.72 (1.61)	0.209	5.41 (1.73)
Serum insulin levels after 60 min	492.85 (391.23)	474.48 (350.54)	0.859	484.19 (369.15)
Homa (mean)	12.02 (6.53)	9.48 (4.20)	0.103	10.82 (5.65)
Abdominal subcutaneous adipocyte size (mean), μm	96.16 (12.78)	94.92 (10.44)	0.702	95.57 (11.64)

*Plus-minus values are means ± SD.

^†^Concentrations of glycated hemoglobin were based on values from the international federation of clinical chemistry and laboratory medicine.

The table presents baseline characteristics for clinical variables and radiological examinations in first-degree relatives and control subjects without diabetes. The complete baseline characteristics table is presented in Supplementary Table 1.

In a next step, we compared imaging and metabolomics data between FDR and study participants without heredity for T2D, henceforth referred to as *control subjects*. We observed no significant differences in distribution of total body fat, abdominal subcutaneous fat, visceral fat, and ectopic fat in liver or heart. In addition, we constructed a generalized linear model, adjusted for age, body mass index and *group* (i.e. FDR or control subjects), which showed no significant differences between baseline characteristics, with the exception of age and 3-MOB, where a p-value of less than 0.05 was considered to indicate statistical significance (see Supplementary Table 2 for more information).

These findings, in conjunction the machine learning models, justified our decision in pooling the imaging and metabolomics data between FDR and control subjects. Nonetheless, we performed sensitivity analyses by constructing separate prediction models for FDR and control subjects, as reported later.

### Magnetic resonance spectroscopy – Liver lipids

**Figure 1:**
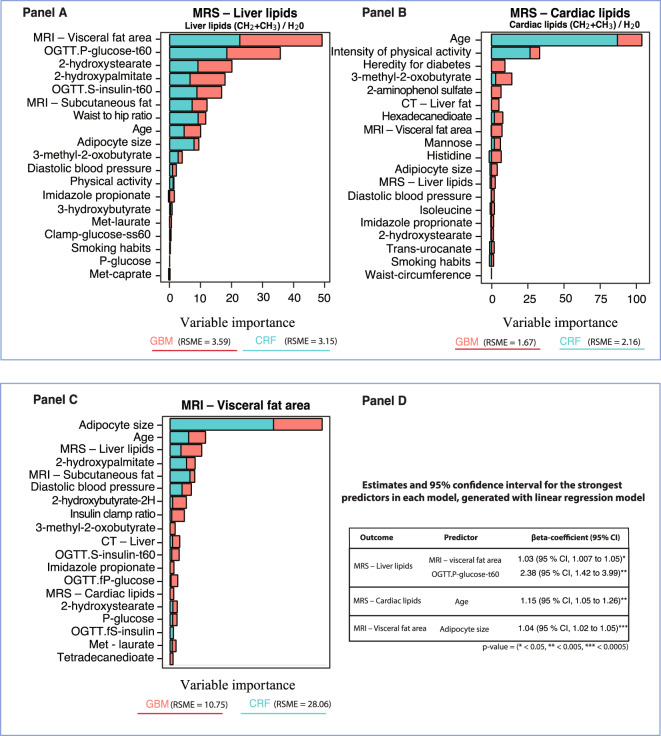
Conditional random forest and gradient boosting for radiological examinations of the lipid accumulation in liver, heart and visceral fat – Relative variable importance by mean decrease accuracy and relative influence These figures (Panels A–C) display the relative importance of phenotype-, imaging- and metabolomic markers for distribution of visceral and ectopic fat according to magnetic resonance spectroscopy- and imaging, using predictive machine learning models. Predictors that display a pronounced increase in relative importance (MDA or relative influence) compared to other predictors are strong predictors for the outcome. The strongest predictors identified from the machine learning models were included in generalized linear regression (Panel D) to demonstrate the risk for each unit increase for the most important predictors.

The data for liver lipid accumulation was first analyzed with conditional random forest, a machine learning model that included all phenotype-, imaging-, protein and serum metabolomics data. As shown in Fig. 1 Panel A, the strongest predictor of liver lipids was amount of visceral fat followed by markers of degree of glucose tolerance, two of the metabolomics markers and insulin response despite the fact that all subjects were non-diabetic/IGT.

Gradient boosting model demonstrated similar results as conditional random forest; i.e.; that visceral fat is the strongest predictor followed by the metabolomics markers 2-hydroxypalmitate and 2-hydroxystearate, as markers of lipid oxidation. Also, the marker of glucose tolerance, expressed as glucose levels at 60 min was a strong predictor, see Fig. 1 Panel A.

Liver fat according to computer tomography also revealed that 2-hydroxybutyrate/2-hydroxyisobutyrate as markers of lipid oxidation were the most important predictors, followed by visceral fat and imidazole propionate, see Fig. 1 Panel C.

**Figure 2:**
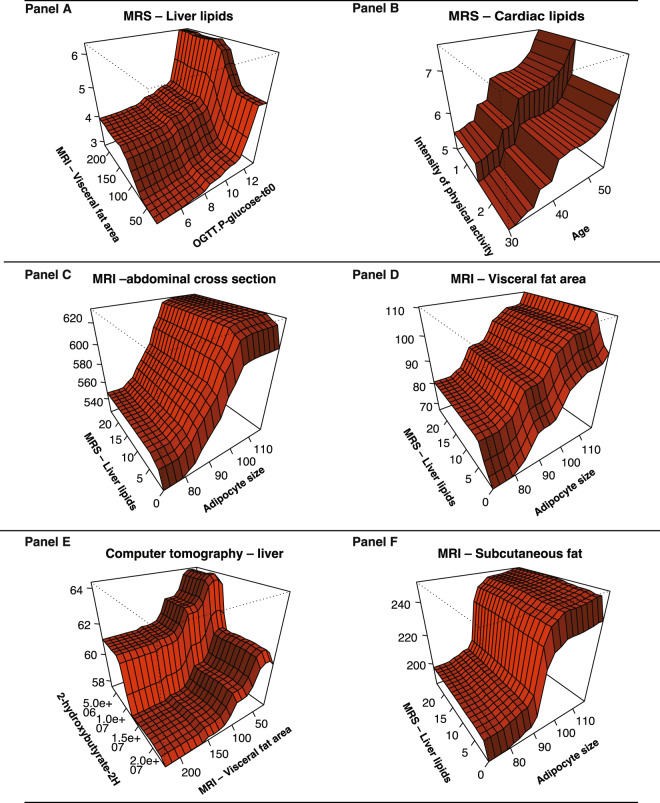
Random forest models for radiological examinations of liver, heart, visceral- and subcutaneous fat and abdominal cross section – Partial dependence plots for strongest predictors These figures (Panels A–F) are two-way partial dependence plots that show the dependence between radiological examinations of fat distribution and the most important predictors, marginalizing over the values of all other features. These are generated with conditional random forest models and enable us to visualize interactions among predictors.

Partial dependence analysis (Fig. 2 Panel A) showed that increasing levels of visceral fat and marker of glucose tolerance were strong interacting factors. The partial dependence plot revealed that the interaction between markers of insulin sensitivity/resistance and visceral fat took place first at higher levels.

Linear regression validated that increasing levels for visceral fat (RR 1.03; 95% CI, 1.007 to 1.05) and OGTT glucose t-60 (RR 2.38; 95% CI, 1.42 to 3.99) were associated with stronger predictability for liver lipids, see Fig. 1 Panel D.

### Magnetic resonance spectroscopy – Cardiac lipids

MR spectroscopy of the heart (Fig. 1 Panel B) showed for the amount of cardiac lipids that age was the strongest individual predictor followed by intensity of physical activity. The conditional random forest model and gradient boosting analysis showed relatively diverse results. However, the gradient boosting model had lowest RSME score at 1.67. Age was a strong predictor in the gradient boosting model followed by 3-methyl-2-oxobutyrate (3-MOB) as the strongest metabolomic predictor, and heredity for diabetes. As shown later, 3-MOB was also the strongest predictor of family history for T2D. The conditional random forest model validated the predictive effect of age and physical activity level, Fig. 1 Panel B. In order to properly assess the importance of heredity for diabetes, we constructed additional machine learning models with a binomial predictor for heredity called *group* (i.e. first-degree relatives vs. control subject), which resulted in similar outcomes.

The partial dependence analyses (Fig. 2 Panel B) revealed minor interactions between the most important predictors for lipid accumulation in heart, according to the machine learning models. However, heredity for diabetes demonstrated strong interaction effects between age and visceral fat. Thus, like liver lipids, cardiac lipids were predicted by amount of visceral fat but physical activity and age were unexpectedly strong predictors. Linear regression showed that only age was a significant predictor for lipid accumulation in heart (RR 1.15; 95% CI, 1.05 to 1.26), Fig. 1 Panel D.

### Magnetic resonance imaging – Visceral fat area

Visceral fat area (Fig. 1 Panel C) was most strongly associated with other adipose tissue stores, in particular subcutaneous adipose cell size and amount followed by age and liver lipids while the strongest metabolomics predictor was again 2-hydroxypalmitate, a marker of lipid oxidation.

Partial dependence analysis (Fig. 2 Panel D) showed that abdominal subcutaneous adipocyte size and amount of liver lipids were strong interacting predictors.

**Figure 3:**
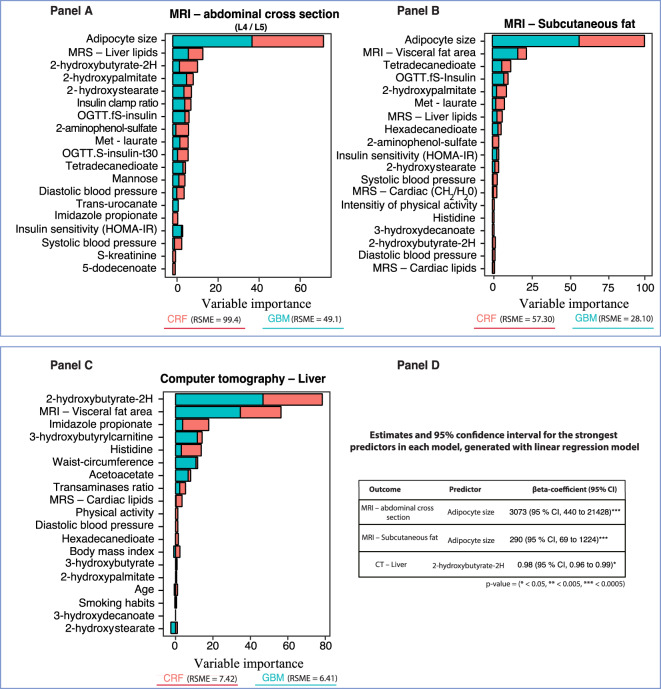
Conditional random forest and gradient boosting for radiological examinations of an abdominal cross section, subcutaneous fat and liver fat according to magnetic resonance imaging and computer tomography – Relative variable importance by mean decrease accuracy and relative influence. These figures (Panel A–C) display the relative importance of phenotype-, imaging- and metabolomic markers for distribution of tissue in abdominal cross section, subcutaneous fat and liver fat according to magnetic resonance imaging and computer tomography. Strongest predictors were identified with machine learning models. Predictors that display a pronounced increase in importance compared to other predictors are strong predictors for the outcome. The regression estimates in Panel D shows the strongest predictors from the random forest models associated with risk increase and which are subsequently included in linear regression models to demonstrate the risk for each unit increase for those predictors.

Gradient boosting (Fig. 1 Panel C) validated the strong impact of adipocyte size, liver lipids and age. These findings were confirmed in predictive models for MRI – abdominal cross section (L4/L5) as seen in Fig. 3 Panel A. Thus, these data show that ectopic fat accumulation in the liver, subcutaneous adipose cell size and markers of lipid oxidation such as 2-hydroxybutyrate/2-hydroxyisobutyrate, are important predictors of each other. Linear regression for visceral fat showed that age was the only significant predictor (RR 1.04; 95% CI, 1.02 to 1.05).

### Magnetic resonance imaging – Abdominal subcutaneous fat

In agreement with the analysis above, amount of subcutaneous adipose tissue was best predicted by subcutaneous adipocyte size followed by visceral fat, markers of insulin sensitivity (Fig. 3 Panel B), and the fatty acid metabolites tetradecanedioate and 2-hydroxypalmitate were also important predictors (Fig. 3 Panel B). However, the partial dependence analysis revealed that adipocyte size and visceral fat were not strong interacting predictors (Fig. 2 Panel F) and also in the gradient boosting analysis (Fig. 3 Panel B). The strongest metabolomics markers were 2-hydroxypalmitate and the dicarboxyl fatty acid tetradecaneoates which have previously been shown to be markers of elevated blood pressure and all-case mortality in the Twins UK and KORA cohorts and also to have effects on blood pressure in animal models^11^.

### Heredity of type 2 diabetes

**Figure 4:**
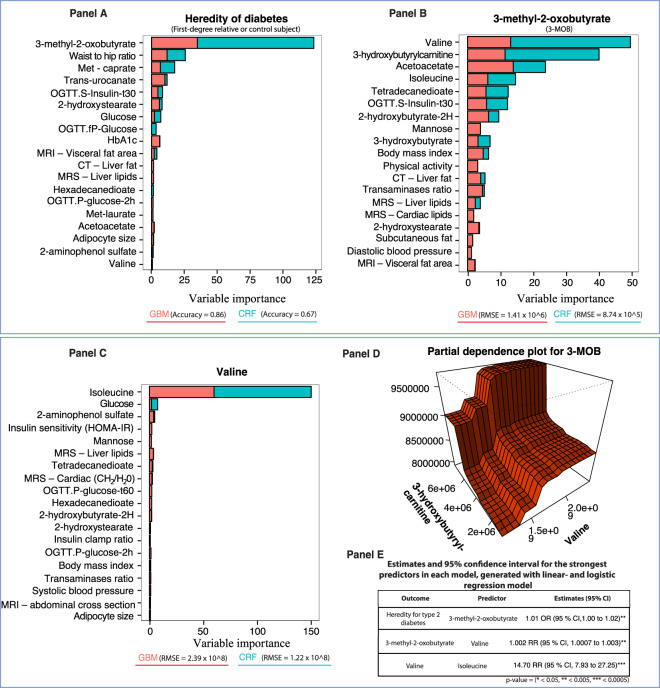
Conditional random forest and gradient boosting models for heredity of type 2 diabetes with stepwise modeling for the strongest predictor in preceding model – Relative variable importance by mean decrease of accuracy and relative influence. These figures (Panels A–D) display the relative importance of phenotype-, imaging- and metabolomic markers for heredity of type 2 diabetes and the predictors that are strongest in each preceding machine learning models as a stepwise modeling approach to identify the metabolite pathway. Predictors that display a pronounced increase in relative importance (MDA or relative influence) compared to other predictors are strong predictors for the outcome. The beta coefficients (95% confidence intervals) next to the strongest predictors in the random forest models are generated from generalized linear regression models, in order to demonstrate the risk for each unit increase. The asterisk (*) denotes that decreasing values for that predictor is associated with risk increase.

We also performed predictive machine learning models for heredity of T2D since the cohort included 25 first-degree relatives. The random forest and gradient boosting models demonstrated that 3-MOB closely followed by waist-hip ratio and caprate, which is an ester of decanoic acid, were the strongest predictors for heredity of diabetes (Fig. 4 Panel A). Stepwise modeling of the most important predictor in the previous model demonstrated that branched-chain amino acids (valine and isoleucine), acetoacetate and insulin sensitivity (OGTT S-Insulin t-30) were the strongest predictors of 3-MOB (Fig. 4 Panel B). The metabolite 3-hydroxybutyrylcarnitine, which is associated with insulin resistance, type 2 diabetes and fatty acid oxidation in the liver, was also an important predictor of 3-MOB and acetoacetate (Fig. 4 Panel B). Partial dependence plots revealed interaction effects between branched-chain amino acids and markers of lipid metabolism, such as the interaction between 3-hydroxybutyrylcarnitine and valine (Fig. 4 Panel D).

### Subcutaneous adipose cell size and insulin sensitivity (HOMA2-IR)

**Figure 5:**
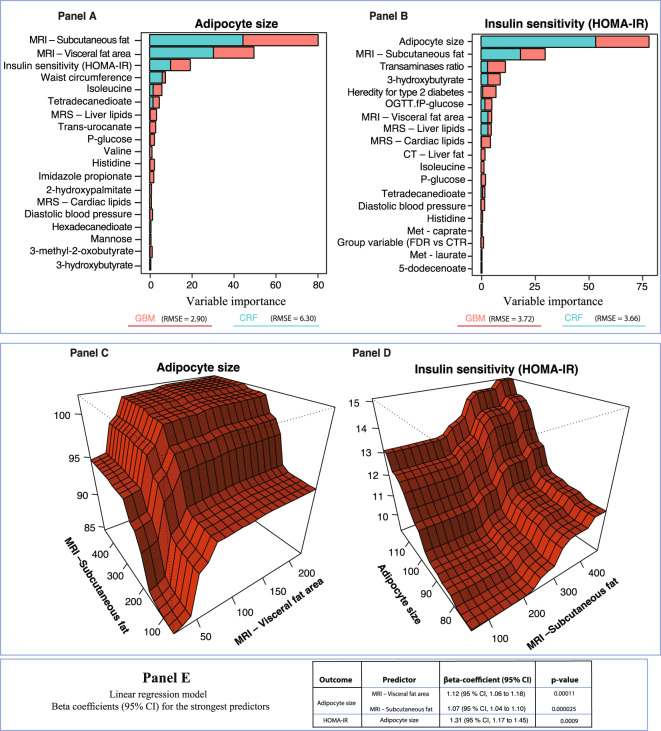
Conditional random forest, gradient boosting and partial dependence plots for adipocyte size and HOMA2-IR, with linear regression – Relative variable importance by mean decrease accuracy. Panels A to B display the relative importance of phenotype-, imaging- and metabolomic markers for adipocyte size and insulin sensitivity (HOMA), using machine learning models. Predictors that display a pronounced increase in importance compared to other predictors are strong predictors for the outcome. Panels C to D show partial dependence plots for the most important predictors identified by predictive machine learning models. Panel E shows risk increase for each unit increase according to linear regression models. *Group variable denotes a binomial predictor for heredity of type 2 diabetes, i.e., either first-degree relative or control subject.

Adipose cell size is rarely measured in clinical studies but appeared as a strong predictor of cardiometabolic risk factors including amount of visceral fat and liver lipids (see Fig. 1 Panels A–C). Thus, we set out to identify predictors of adipose cell size. As shown in Fig. 5 Panels A–B, fat in visceral and subcutaneous depots were strong predictors followed by measures of insulin sensitivity and waist circumference. We constructed additional models as sensitivity analyses to evaluate the strongest predictor for abdominal subcutaneous adipocyte size in a model without imaging variables and waist circumference. This revealed that insulin sensitivity (euglycemic clamp data) was the most important predictor closely followed by mono- and dicarboxyl fatty acids such as trans-urocanate and tetra-decanedioates as the strongest metabolomics markers of subcutaneous adipose cell size.

Furthermore, we scrutinized predictors of HOMA2-IR; a widely used clinical marker of insulin sensitivity. The initial machine learning models revealed¸ as expected, that glucose tolerance and insulin sensitivity were the strongest predictors since they are used to construct the HOMA index. These were, therefore, excluded from the analysis. Under these conditions, subcutaneous adipocyte cell size was the most important predictor for HOMA2-IR and explained approximately 40–60% of overall predictability, Fig. 5 Panel B. The conditional random forest model for HOMA2-IR, not including radiological examination and serum insulin after oral glucose tolerance test, demonstrated similar importance for subcutaneous adipocyte cell size. 3-hydroxybutyrate was also strongly related to HOMA2-IR. Taken together, subcutaneous adipose cell size is an integrated marker of amount of ectopic fat and adipose tissue mass and, as such, an excellent marker of whole-body inulin sensitivity.

### Relative contribution of imaging data and heredity for type 2 diabetes

In addition to the relative importance of each predictor, we also calculated the relative contribution, i.e. the percentage of each predictor explained in every machine learning model and, thereafter, calculated the average of these percentages for each predictor from the random forest and gradient boosting models.

**Figure 6:**
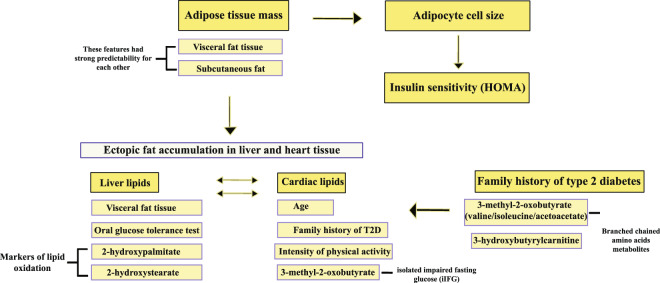
Relative importance between imaging, phenotype and metabolomics data, in individuals with- and without heredity for type 2 diabetes. The relative importance for predictors was ranked according to highest relative contribution of each predictor in both the random forest model and gradient boosting model. The size of the arrows does not indicate greater strength between the predictors and outcomes.

Figure 6 displays a mapped network between these predictors and how they are intertwined based on the relative contribution. Our machine learning analyses show that visceral fat, subcutaneous adipose tissue and waist-hip ratio are all strong predictors of adipose tissue mass and subcutaneous adipose cell size which, in turn, is a strong predictor for insulin sensitivity (HOMA2-IR).

Moreover, adipose tissue mass, most strongly visceral fat, is an important predictor for ectopic fat distribution in liver and heart. The metabolite 3-methyl-2-oxobutyrate (3-MOB), shown to be a marker of impaired fasting glucose (IFG)^12^ and a strong predictor of ectopic fat accumulation. For heredity of type 2 diabetes, 3-MOB was a strong predictor along with glucose tolerance and insulin sensitivity. We performed stepwise modeling, which validated that BCAA and related metabolite (3-MOB) were strong predictors of heredity for type 2 diabetes.

## Discussion

The purpose of this extensive study was to identify predictors of cardiometabolic risk profile based on different imaging analyses, glucose tolerance, insulin sensitivity, family history of diabetes and untargeted serum metabolomics. Family history is well known to be one of the most prominent risk factors for future development of T2D^13^. We sought to eliminate differences in baseline characteristics between control subjects and first-degree relatives. Therefore, all study participants were matched for BMI, blood pressure, waist circumference, fasting glucose and insulin levels, with the exception of age, which was significantly higher in first-degree relatives (6 years) (Table 1). Considering this, the predictor termed ‘first-degree relative’ should not be significantly influenced by metabolic- or body composition differences.

It is clear that amount of *visceral fat* is the best marker of liver lipids, a strong marker of heart lipids and it was also a major marker of amount of subcutaneous adipose tissue and its adipose cell size. We have previously also shown that waist circumference, a well-established marker of visceral fat^14^, is strongly correlated to subcutaneous adipose cell size and the current data corroborate this^15^.

Interestingly, age was strongly associated with both heart, visceral and liver lipids. This is also consistent with previous findings showing that amount of visceral fat increases with age independent of BMI^14^. This increased visceral and ectopic fat accumulation may be caused by aging-associated cell senescence, which prevents the formation of new functional cells in the subcutaneous adipose tissue and leads to lipid spill-over^4,16^. Surprisingly we observed that degree of physical activity was positively related to amount of heart lipids, which is similar to what has been shown for lipids in skeletal muscles^17^. Thus, this may be a general effect of physical activity and unrelated to negative metabolic consequences.

Expansion of subcutaneous adipose cells is strongly associated with reduced whole-body insulin sensitivity, enhanced inflammation and it is also an independent predictor of future risk of developing T2D^2,18,19^. It is a consequence of inability to recruit new functional adipose cells^10^, which, in turn, is associated with a genetic predisposition for T2D, but, not for obesity^6,7^, and increased precursor cell senescence^4^. Several recent studies have also shown that individuals with genetic markers of insulin resistance are characterized by reduced subcutaneous adipose tissu^8,9^, which is expected to increase the adipose cell size and accumulation of ectopic lipids.

Interestingly, we also found that adipose cell size is a major predictor of whole-body insulin sensitivity measured as *HOMA index* or with euglycemic clamps and that visceral fat is a predictor of subcutaneous adipose cell size as well as a marker of glucose tolerance. Alfa-hydroxybutyrate (α-HB) was the strongest metabolomics predictor of HOMA insulin sensitivity. Alfa-hydroxybutyrate has also previously been shown as a marker of insulin resistance, IGT and progression to developing T2D^20,21^.

Glucose tolerance and degree of insulin sensitivity were strong predictors of *family history of T2D*. However, the strongest predictor was 3-methy-2-oxobutyrate (3-MOB), a key metabolite of branched-chain amino acid (BCAA)^22^ and isoleucine as well as valine (not shown) were also strong predictors. We also found mannose as a marker of insulin resistance and future risk of developing both T2D and CVD^23,24^ to be a predictor of family history and its phenotype. Detailed partial dependence plots also showed that isoleucine and a-HB strongly interacted to predict 3-MOB levels and this was also found for valine (data not shown). Previous work has shown that a-HB levels predict IGT while 3-MOB is more closely associated with IFG^20^. However, all our subjects had normal GTT and no IGT or IFG were included. This suggests that these biomarkers of family history actually predict inherent factors associated with family history and future risk of developing glucose intolerance.

Amount of *liver lipids* and *heart lipids* showed quite distinct differences in their predictors. Amount of visceral fat, lipid oxidation products, glucose tolerance and amount of abdominal subcutaneous fat and adipose cell size mainly predicted liver lipids. Heart lipids were also predicted by amount of visceral fat and liver lipids, but more strongly by age and degree of physical activity as discussed. In fact, age was an important predictor of amount of visceral fat as well as ectopic fat. This is likely a consequence of increased subcutaneous adipose tissue progenitor cell senescence^4,16^ preventing recruitment of new cells and promoting lipid storage in other depots.

The strength of this study is the extensive phenotyping together with imaging data and metabolomics markers while a weakness is the limited number of individuals included. However, most previous studies have not been able to integrate all information as generated in this study. In Fig. 6, we have summarized our key findings of predictors and their apparent associations. It is clear that amount of adipose tissue, also in this non-obese cohort, and subcutaneous adipose cell size are central factors associated with both insulin sensitivity (HOMA and clamp data) and ectopic liver lipids. In fact, the most prominent predictor of HOMA, excluding glucose and insulin levels, was adipose cell size while a-HB was the most prominent metabolomics marker. Increased subcutaneous adipose cell size is associated with amount of adipose tissue and, in particular, visceral fat and waist circumference. Our study comprised exclusively of non-obese men, therefore, it is conceivable that these findings are imprecise for women.

Taken together, our current data show that subcutaneous adipose tissue mass and cell size, even in the non-obese state, are key predictors of whole-body insulin sensitivity as well as visceral and ectopic hepatic lipid accumulation. Family history of T2D is predicted by both OGTT and HOMA, but the most prominent marker was the BCAA metabolite 3-MOB as well as mannose and a-HB. It is likely that family history of T2D and its associated genetic effects are central for both the expanded adipose cell size and accumulation of ectopic fat, which are all components of the cardio-metabolic risk profile.

## Study Population and Methods

The local Ethical Committee at the Sahlgrenska Academy at the University of Gothenburg approved the study protocol. The study was performed in agreement with the Declaration of Helsinki. All subjects received oral and written information and gave their informed consent to participate.

### Study population

We recruited in total 25 first-degree relative and 28 control persons via newspaper advertisements and through earlier studies performed at the laboratory. Inclusion criteria in the first-degree relative group were male sex, at least one first-degree relative with a diagnosis of type 2 diabetes mellitus and general good health. Inclusion criteria in the control group were male sex, no first-degree relative with type 2 diabetes mellitus and general good health. Most recruited individuals had previously undergone examinations in the laboratory and had gained around 8% in body weight since previous investigations. We also recruited other individuals from previous recent investigations and they were placed on a supervised hypercaloric diet to also increase body weight with around 8% before inclusion in the study. This was done to ensure similar changes in body weight and hypercaloric diet intake in the groups. Subcutaneous adipose tissue biopsies were performed from the lower abdominal wall and processed for cell sizing as previously reported^10^. This study was approved by the regional ethical committee (approvals 384-12 and T803-13). All general clinical investigation methods have been described previously^25,26^.

### Clinical variables

Lifestyle factors were evaluated through a questionnaire filled out at the laboratory. Body weight and height, and waist and hip circumferences were recorded and BMI was calculated. The proportions of body fat and lean body mass were determined using bioelectrical impedance (single frequency, 50 kHz; Animeter, HTS, Odense, Denmark). Blood pressure was measured in a sitting position after a five minutes rest with a mercury sphygmomanometer.

To evaluate glucose tolerance status, fasting blood samples were drawn after 12 hours of fasting and were followed by an OGTT (75 g glucose orally). Samples for measurement of plasma glucose and serum insulin were drawn after 0, 30, 60 and 120 minutes.

Again after 12 hours of fasting, an intravenous glucose tolerance test (IVGTT) was performed to determine the first and second phases of insulin secretion. A bolus of glucose (300 mg/kg in a 50% solution) was given within 30 seconds into the antecubital vein. Samples for the measurement of plasma glucose and insulin (arterialized venous blood) were drawn at −5, 0, 2, 4, 6, 8, 10, 20, 30, 40, 50 and 60 minutes. The acute and the late insulin responses, i.e. incremental area under the insulin curve, (AIR, 0-10 minutes; LIR, 10-60 minutes) were calculated using the trapezoidal method.

In subgroup 1 the IVGTT was followed by a hyperinsulinemic euglycaemic clamp was initiated (insulin infusion: 240 pmol m^−2^ min^−1^ for 120 min) to evaluate insulin sensitivity as previously reported^27^. Whole blood glucose was clamped at 5.0 mmol/l for the next 120 minutes by infusion of 20% glucose at various rates according to glucose measurements performed at five minutes intervals (YSI, Yellow Springs Instrument Company, OH). Insulin sensitivity (M) was calculated as the mean glucose infusion rate during the last 30 minutes of the clamp adjusted for body weight, and M/I was calculated as the M-value corrected for steady-state insulin concentrations. In all subgroups, fasting plasma insulin and fasting plasma glucose from the OGTT were used to calculate a HOMA-IR index using the formula HOMA-IR = (fasting plasma glucose × fasting plasma insulin)/22.5 M and M/I were used to validate the HOMA-IR. Only the HOMA-IR value is reported here to assess insulin sensitivity. All metabolites were measured in serum subsequent to overnight fasting.

Plasma glucose was measured using standard laboratory methods (Department of Chemistry, Sahlgrenska University Hospital, Gothenburg, Sweden). Plasma insulin was measured at the University of Tübingen, Germany, by micro-particle enzyme immunoassay (Abbott Laboratories, Tokyo, Japan).

A subcutaneous abdominal adipose tissue biopsy was performed. The biopsies (approximately 20–30 mg) were obtained with a needle aspiration technique, from the paraumbilical region after local infiltrative anesthesia with lidocaine (20 ml, 0.5%)^28^.

Isolation of adipocytes was performed by initially washing the biopsied to remove traces of blood, followed by and treatment with collagenase (1 mg/ml) (Sigma, St Louise, MO, USA) for 60 minutes at 37 °C in a shaking water bath. Isolated adipocytes were filtered through a 250 mm nylon mesh and washed with fresh medium. Adipocyte cells were placed on a siliconized glass slide and 100 consecutive cell diameters were measured with a calibrated ocular and expressed as the average value in μm.

### MRI and MRS for fat determination

MR imaging and localized ^1^H-MR spectroscopy was performed using a 1.5 T MR-system (Intera/Achieva, software release 3.2) using the vendor´s 16 channel SENSE XL Torso coil (Philips Medical Systems, Best, The Netherlands). The software used included a research package enabling navigator triggered MRS and a field map based B_0_-shimming^29,30^.

### MRI to assess abdominal fat

The amount of intra-abdominal and subcutaneous fat was assessed at the level between the 4th and the 5th lumbar vertebra (L4/L5) using T1 weighted axial images acquired with a TE of 5.24 ms, TR of 91 ms, 80 degree flip angle, pixel size of 1*1 mm^2^ and a 10 mm slice thickness.

Data processing was performed using an in-house developed segmentation program written in MatLab (MATLAB R2014b, The MathWorks Inc, USA). An intensity threshold value for fat signal was determined for each individual patient and the surface of intra-abdominal and subcutaneous adipose tissue was quantified. Inter-muscular fat, as well as muscle, bone and lean tissue was excluded. The fat fraction is reported as intra-abdominal to total volume, and subcutaneous adipose tissue to total volume ratios.

### MRS to asses liver fat

Liver spectra were acquired in end expiration using point resolved spectroscopy (PRESS) with a TE of 30 ms and TR of 2700 ms. Four non-water suppressed signals and 32 water suppressed (CHESS) signals were acquired with 1.0 kHz spectral bandwidth and 1024 data points. The voxel (2.5 × 2.5 × 2.5 cm^3^) was positioned within the right liver lobe and care was taken to exclude large intrahepatic blood vessels, bile ducts and abdominal adipose fat.

The liver MRS data were processed using the jMRUI software. In jMRUI the spectra were eddy current corrected, and residual water and base line were removed using a Hankel-Lanczos filter (HLSVD, a single decomposition method). For the non-water suppressed reference spectra all metabolite signals, except water, were removed using the HLSVD algorithm. In the processed spectra water (H_2_O, 4,7 ppm), methylene (CH_2_, 1.3 ppm) and methyl (CH_3_, 0.9 ppm) were quantified using the AMARES algorithm and all metabolites were corrected for T2 relaxation using T2 values from the literature^31–33^. The fat fraction(%), denoted MRS-liver lipids in results, is calculated as (CH_2_ + CH_3_)/H_2_O.

### MRS to assess heart lipids

Cardiac MRS measurements were performed using PRESS with a TE of 35 ms. The spectroscopy scans were cardiac triggered to end systole, using individually optimized time-delays^33^, and respiratory triggered at end expiration. Eight non-water suppressed dynamics (TR = 6000 ms) and 128 water suppressed (CHESS) dynamics (TR = 3000 ms) were acquired. The voxel (4.5cm^3^) was carefully planned within the ventricular septum and care was taken to minimize effects from blood and epicardial lipid contamination.

The cardiac MRS data were processed using the jMRUI software. In jMRUI the spectra were eddy current corrected and residual water and base line were removed using HLSVD. In the processed spectra trimethylamines (TMA), creatin (Cr), methylene (CH_2_), methyl (CH_3_) and an additional lipid complex at 2.1 ppm were quantified using the AMARES algorithm. All metabolites were corrected for T2 relaxation using T2 values from the literature and the fat fraction (%), denoted as MRS cardiac lipids in results, was calculated as (CH_2_ + CH_3_)/H_2_O.

### Statistical analysis

Our analysis of data involves baseline characteristics for phenotype-, imaging- and metabolomic variables for all study participants and according to hereditary status for diabetes, presented as mean ± SD (Table 1 and supplementary Table 1). Correlation between covariates in persons with first-degree relatives and control subjects were tested by Spearman correlation coefficient (supplementary figure 1). A general linear model were applied to test for associations while controlling for the potential confounders, i.e. body mass index, age and a variable termed *group*, denoting first-degree relative or control subjects, see supplementary Table 2 for more details.

### Predictive models

We constructed predictive models with Conditional Random forest and Gradient boosting to examine relative variable importance, i.e. predictive ability of a broad range of predictors for amount of ectopic-, visceral- and abdominal subcutaneous fat accumulation, assessed with various radiological examinations. Random forest and Gradient boosting are both machine learning algorithms.

### Conditional random forest

The random forest models were based on *conditional* inference trees, in order to reduce bootstrap sample bias and variable selection bias, resulting in more accurate measurements of variable importance, particularly for categorical predictors^34,35^. Random forest is a nonparametric machine learning model that consists of a collection of decision trees with random feature selection. Each regression tree is tested on a bootstrap sample of two-third of the original dataset for training instances, features and one-third for out-of-bag (OOB) measures. Conditional random forest has built-in mechanisms for estimation of test error and certainty in each prediction model by using OOB-error rate. Initially, internal-validation mechanism was utilized to construct conditional random forest models that minimalized the OOB-error rate. Thereafter, results from internal validation techniques were compared to k-fold cross validation with test and training dataset, using 3 to 5 iterations for various models turned out to be superior.

### Gradient boosting models

In addition to conditional random forest models, we constructed gradient boosting models to compare results from various models. Gradient boosting is another machine learning model that constructs ensembles of decision trees and measures variables importance in similar ways as conditional random forest models. However, the prediction is performed differently. In Gradient boosting each tree is grown on the residuals of the previous tree and prediction is subsequently accomplished by weighting the ensemble outputs of all the regression trees. In the gradient boosting models, we assumed Gaussian distribution for reducing the squared-error loss and the shrinkage factor applied to each tree was set to 0.001^36^. With these settings, the gradient boosting models showed rather similar predictions as the conditional random forest models.

### Model building

Hyperparameter optimization was performed for every machine learning model, using automated grid search. Parameters that were evaluated in the conditional random forest model included number of trees, predictors in each split, minimum sum of weights in a node to be considered for splitting and the proportion of observations needed to establish a terminal node. Machine learning models were based on hyperparameters that minimize root mean squared error (RSME). Grid search for the gradient boosting models included the parameters interaction depth, number of trees, shrinkage factor and number of observations in a terminal node.

Each conditional random forest models contains a unique subset of predictors since features with near-zero influence (negative MDA-score) and correlated predictors were excluded in the preliminary machine learning models. Feature selection for each model was based on subject matter knowledge, to improve signal extraction of predictors, improve model diagnostics and metrics (RSME). In the gradient boosting models, predictors with low relative influence were sequentially excluded until relative importance plot demonstrated stability for the strongest predictors. Each random forest and gradient boosting model were built using between 600 to 3,500 regression- or classification trees. Additional model diagnostics was performed with measurement of r-squared (R^2^) on test sets for each model. However, due to small sample size, we noticed relatively large fluctuations in R^2^ depending on partition size of test samples. Therefore, model building was performed to minimize RMSE and optimize R^2^ values for each model.

Relative importance of predictors is presented as mean decrease of accuracy (MDA) and relative influence for conditional random forest and gradient boosting, respectively, a large relative importance indicates that the predictor is important, whereas a small MDA or relative influence value indicates that the predictor is less important for that outcome.

### Partial dependence plots

Comprehensive model building revealed interactions between strong predictors for certain outcomes. Therefore, we constructed two-way partial dependence plots (Figs. 2, 4 and 5), based on random forest models, which demonstrates the interaction effects of varying values for the most important predictors. Partial dependence plots visualizes the relationship between predictors, whether it is linear, monotonous, more complex or if it’is increasing or decreasing values that is related with the outcome^37^.

### Linear- and logistic regression models

The most important predictors identified by means of machine learning models were subsequently included in linear and logistic regression models. The regression estimates and 95% confidence intervals that are presented in Fig. 1 Panel D, Fig. 3 Panel D, and Figs. 4–5 Panel E. These were generated from regression models using log-transformed variables and unstandardized regression coefficients. These models were intended to validate the partial dependence plots to ensure that a decrease or increase in a feature value was associated with increased predictability of the outcomes.

### Imputation

We used missForest package in R to impute missing data for study participants, this package is based on the random forest algorithm^37^. We analyzed distributions and means before and after imputation without observing virtually any differences. A p-value of less than 0.05 were considered to indicate statistical significant.

Calculations were performed in R (v 3.6.2) using the following packages: Corrplot, GBM, missForest, Random Forest, Caret, ggRandomForests, Party, Plotmo, GridExtra, cForest, MLR and hydroGOF. R package version 2.25).

## Supplementary information

Supplementary material.

## Data Availability

All computer code used to generate results reported in the manuscript will be available to editors upon request. The data is available on a general public repository (www.datadryag.org), submitted with 10.5061/dryad.4qrfj6q7b.
