# Supplementary material for: Adipose tissue morphology, imaging and metabolomics predicting cardiometabolic risk and family history of type 2 diabetes in non-obese men
Source: Sci Rep. 2020 Jun 19;10:9973. doi: 10.1038/s41598-020-66199-z (PMC7305301; doi:10.1038/s41598-020-66199-z)
Supplement: Supplementary file 1 — Supplementary material. [file 41598_2020_66199_MOESM1_ESM.docx]

# Adipose tissue morphology, imaging and metabolomics predicting cardiometabolic risk and family history of type 2 diabetes in non-obese men

**Supplemental material**

**Authors:** Aidin Rawshani^1a^, Björn Eliasson^1a^, Araz Rawshani^1^, Josefin Henninger^1^, Adil Mardinoglu^2,3^, Åsa Carlsson^4^, Maja Sohlin^4^, Maria Ljungberg^4^, Ann Hammarstedt^1^, Annika Rosengren^1^ And Ulf Smith^1*^

**Affiliations:**

^1^ The Lundberg Laboratory for Diabetes Research, Department of Molecular and Clinical Medicine, The Sahlgrenska Academy at the University of Gothenburg, Gothenburg, Sweden.

^2^ Science for Life Laboratory, KTH - Royal Institute of Technology, Stockholm, Se-17121, Sweden

^3^ Centre for Host-Microbiome Interactions, Faculty of Dentistry, Oral & Craniofacial Sciences, King's College London, London, Se1 9rt, United Kingdom

^4^ Institute of Clinical Sciences, Dept. of Radiation Physics, Sahlgrenska Academy at the University of Gothenburg, Gothenburg, Sweden.

^A^ Contributed equally

* Corresponding author.

E-mail address: Ulf.Smith@medic.gu.se

**One sentence summary:** Increased subcutaneous adipose tissue cell size is a marker of whole-body insulin sensitivity and ectopic fat accumulation while 3-methyl-2-oxobutyrate and BCAA metabolites are predictors of heredity of diabetes.

# TABLE OF CONTENT

TABLE OF CONTENT 2

Supplementary Table 1. Baseline characteristics for clinical variables and radiological examinations in first-degree relatives and control subjects without diabetes 3

Supplementary Table 2. Generalized linear model of predictors adjusted for age, body mass index and *group* in individuals with first degree relative with diabetes and control subjects 5

Supplementary Figure 1. Correlations matrix for predictors included in the analyses 7

| Supplementary Table 1. Baseline characteristics for clinical variables and radiological examinations in first-degree relatives and control subjects without diabetes | | | | |
| --- | --- | --- | --- | --- |
| **Characteristics** | **Control subjects** | **First-degree relatives** | **P-value** | **Overall** |
| Number | 28 | 25 |  | 53 |
| Age – yr | 39.29 (7.95)* | 45.60 (7.34) | 0.004 | 42.26 (8.23) |
| **Blood pressure – mmhg** | | | | |
| Diastolic blood pressure | 79.54 (9.34) | 82.20 (10.70) | 0.338 | 80.79 (9.99) |
| Systolic blood pressure | 125.86 (12.51) | 128.92 (11.98) | 0.368 | 127.30 (12.24) |
| Body mass index, kg/m^2^ | 25.26 (3.88) | 25.93 (3.12) | 0.492 | 25.57 (3.52) |
| Glycated hemoglobin (mmol/mol)† | 32.50 (2.42) | 33.31 (2.16) | 0.207 | 32.88 (2.31) |
| Waist to height (mean) | 88.77 (10.89) | 92.00 (7.83) | 0.226 | 90.29 (9.62) |
| Serum creatinine, μmol/L | 90.46 (11.37) | 86.12 (9.09) | 0.134 | 88.42 (10.49) |
| Waist to hip ratio | 0.87 (0.06) | 0.90 (0.05) | 0.090 | 0.89 (0.06) |
| **Oral glucose tolerance test (OGTT)** | | | | |
| Fasting plasma glucose, mmol/L | 4.86 (0.43) | 5.01 (0.39) | 0.211 | 4.93 (0.41) |
| Fasting serum insulin, pmol/L | 47.82 (25.49) | 42.64 (18.11) | 0.403 | 45.38 (22.26) |
| Plasma glucose levels after 60 min | 7.71 (2.32) | 7.72 (1.87) | 0.983 | 7.71 (2.10) |
| Plasma glucose levels after 2h | 5.12 (1.82) | 5.72 (1.61) | 0.209 | 5.41 (1.73) |
| Serum insulin levels after 60 min | 492.85 (391.23) | 474.48 (350.54) | 0.859 | 484.19 (369.15) |
| HOMA (mean) | 12.02 (6.53) | 9.48 (4.20) | 0.103 | 10.82 (5.65) |
| Adipocyte size (mean) | 96.16 (12.78) | 94.92 (10.44) | 0.702 | 95.57 (11.64) |
| **Radiological examinations** | | | | |
| Computer tomography – liver fat | 60.74 (11.73) | 61.18 (8.73) | 0.879 | 60.94 (10.33) |
| MRI – Visceral fat area (L4/L5) | 83.33 (48.80) | 90.04 (46.05) | 0.610 | 86.49 (47.18) |
| MRI – Subcutaneous fat | 227.06 (102.91) | 215.18 (71.65) | 0.632 | 221.46 (88.90) |
| MRI – abdominal cross section (mean) | 593.15 (146.51) | 585.69 (107.45) | 0.835 | 589.63 (128.41) |
| MRS – Liver lipids | 3.28 (5.28) | 3.53 (3.50) | 0.843 | 3.39 (4.49) |
| MRS – Cardiac lipids | 5.45 (2.49) | 6.51 (3.31) | 0.192 | 5.95 (2.93) |
| **Hereditary status for type 2 diabetes – no. (%)** | | | | |
| 0 = no relatives with diabetes | 28 (100.0) | 0 (0.0) |  | 28 (52.8) |
| 1 = 1 FDR | 0 (0.0) | 13 (52.0) |  | 13 (24.5) |
| 2 = 2 FDR | 0 (0.0) | 4 (16.0) |  | 4 (7.5) |
| 3 =1 FDR and 1 second degree relative | 0 (0.0) | 7 (28.0) |  | 7 (13.2) |
| 4 = 1 FDR or 1 second degree relatives with insulin dependent diabetes mellitus | 0 (0.0) | 1 (4.0) |  | 1 (1.9) |
| **Physical activity – no. (%)** | | | | |
| 1 = Never | 6 (21.4) | 3 (12.0) |  | 9 (17.0) |
| 2 = once per week | 1 (3.6) | 3 (12.0) |  | 4 (7.5) |
| 3 = 2-3 times /week | 6 (21.4) | 13 (52.0) |  | 19 (35.8) |
| 4 = 4-6 times/week | 10 (35.7) | 4 (16.0) |  | 14 (26.4) |
| 5 = every day | 5 (17.9) | 2 ( 8.0) |  | 7 (13.2) |
| Glucose clamp ratio | 0.02 (0.01) | 0.02 (0.00) | 0.903 | 0.02 (0.01) |
| Liver transaminases ratio (ASAT/ALAT) | 0.96 (0.22) | 1.04 (0.42) | 0.351 | 1.00 (0.33) |
| Intensity of physical activity – no. (%) | 21 (75.0) | 13 (52.0) | 0.145 | 34 (64.2) |
| Smoking – no. (%) | 1 (3.6) | 4 (16.0) | 0.283 | 5 (9.4) |
| Insulin clamp (b-glucose 40) | 5.11 (0.11) | 5.14 (0.21) | 0.444 | 5.12 (0.16) |
| Insulin clamp (b-glucose 60) | 5.09 (0.07) | 5.11 (0.14) | 0.573 | 5.10 (0.11) |
| * Plus-minus values are means ± SD  † Concentrations of glycated hemoglobin were based on values from the International Federation of Clinical Chemistry and Laboratory Medicine. | | | | |

| Supplementary Table 2. Generalized linear model of predictors adjusted for age, body mass index and *group* in individuals with first degree relative with diabetes and control subjects | | | | |
| --- | --- | --- | --- | --- |
|  | **Estimate** | |  |  |
| **Characteristics** | **First degree relative (FDR)** | **Control subjects (CTR)** | **p-value** | **Difference** |
| **Blood pressure – mm Hg** | | | | |
| Diastolic blood pressure | 80.56 (76.72-84.41) | 81.00 (77.38-84.62) | 0.87 | 0.43 |
| Systolic blood pressure | 128.04 (123.04-133.03) | 126.65 (121.94-131.35) | 0.69 | 1.39 |
| Body mass index, kg/m^2^ | 25.59 (24.12-27.05) | 25.56 (24.18-26.94) | 0.98 | 0.02 |
| Glycated hemoglobin,mmol/mol | 33.36 (32.42-34.30) | 32.47 (31.59-33.36) | 0.19 | 0.88 |
| Waist circumference, cm | 90.72 (89.10-92.34) | 89.92 (88.40-91.45) | 0.48 | 0.8 |
| Serum creatinine,μmol/L | 86.32 (81.89-90.74) | 90.29 (86.12-94.45) | 0.21 | 3.96 |
| Waist to hip ratio | 0.89 (0.87-90.00) | 0.88 (0.87-9.00) | 0.87 | 0.00184 |
| Weight – kg | 83.39 (80.69-86.09) | 83.95 (81.42-86.49) | 0.76 | 0.56 |
| Age – yr | 45.43 (42.39-48.47) | 39.44 (36.57-42.31) | 0.006 | 5.99 |
| **Oral glucose-tolerance test (OGTT)** | | | | |
| Fasting plasma glucose, mmol/L | 4.95 (4.79-5.12) | 4.92 (4.76-5.07) | 0.76 | 0.03 |
| Fasting plasma insulin,pmol/L | 40.86 (32.72-48.99) | 49.42 (41.76-57.07) | 0.14 | 8.56 |
| Plasma glucose after 60 min | 7.46 (6.59-8.32) | 7.94 (7.13-8.76) | 0.43 | 0.49 |
| Plasma glucose after 2h | 5.57 (4.88-6.26) | 5.26 (4.61-5.91) | 0.53 | 0.31 |
| Serum insulin after 30 min | 344.57 (264.82-424.33) | 444.17 (369.15-519.19) | 0.08 | 99.6 |
| Serum insulin after 60 min | 448.14 (296.75-599.52) | 516.38 (373.98-658.77) | 0.52 | 68.24 |
| Clamp avg (b-glukos-40) | 5.12 (5.06-5.18) | 5.12 (5.06-5.18) | 0.97 | 0.00133 |
| Clamp avg (b-glukos-60) | 5.10 (5.06-5.14) | 5.10 (5.07-5.14) | 0.86 | 0.0047 |
| Clamp avg insulin | 579.87 (549.45-610.30) | 590.05 (561.44-618.67) | 0.63 | 10.12 |
| **Radiological examinations** | | | | |
| Computer tomography – liver fat | 62.18 (58.50-65.86) | 63.27 (59.81-66.73) | 0.67 | 1.09 |
| MRI – visceral fat area | 77.94 (65.90-89.99) | 81.70 (70.37-93.03) | 0.66 | 3.76 |
| MRI – subcutaneous fat | 203.97 (184.78-223.15) | 209.57 (191.52-227.61) | 0.68 | 5.6 |
| MRI – abdominal cross section | 560.41 (540.66-580.15) | 567.59 (549.02-586.17) | 0.61 | 7.19 |
| MRS – cardiac lipids | 6.26 (5.19-7.33) | 5.78 (4.77-6.79) | 0.52 | 0.48 |
| MRS – liver lipids | 2.93 (1.18-4.68) | 3.81 (2.16-5.45) | 0.48 | 0.87 |
| Adipocyte size | 93.38 (89.09-97.67) | 97.41 (93.37-101.44) | 0.19 | 4.02 |
| HOMA | 9.11 (6.98-11.24) | 12.33 (10.32-14.33) | 0.038 | 3.21 |
| Liver fat | 61.84 (57.43-66.26) | 60.24 (56.09-64.40) | 0.61 | 1.6 |
| 5-dodecenoate | 3907222 (3342161-4472284) | 4189962 (3658463-4721462) | 0.48 | 282740 |
| 2-hydroxypalmitate | 39371302 (35973708-4276890) | 37369440 (34173646-4056523) | 0.41 | 2001861 |
| 3-hydroxydecanoate | 1333265 (1128795-1537736) | 1421168 (1228843-1613494) | 0.54 | 87903 |
| 2-aminophenol sulfate | 1103960 (720764-1487158) | 1078872 (718435-1439309) | 0.92 | 25088 |
| 2-hydroxybutyrate-2-hydroxyisobutyrate | 12430648 (10799555-1406174) | 12456199 (10921985-1399041) | 0.98 | 25550 |
| 2-hydroxystearate | 10378971 (9672577-1108537) | 9806046 (9141608-1047048) | 0.25 | 572924 |
| Trans urocanate | 4085601 (3290034-4881170) | 4489123 (3740808-5237439) | 0.47 | 403521 |
| Tetradecanedioate | 537321 (459140-6155034) | 581554 (508016-6550924) | 0.42 | 44232 |
| 3-hydroxybutyrate | 47593943 (30037748-6515014) | 52727612(36214168-6924106) | 0.68 | 5133669 |
| 3-methyl-2-oxobutyrate | 7565196 (6915133-8215259) | 9158955 (8547503-9770408) | 0.0011 | 1593759 |
| Hexadecanedioate | 689697 (589335-7900606) | 766198 (671796-8605998) | 0.28 | 76500 |
| 3-hydroxybutyrylcarnitine | 2499719 (1883274-3116164) | 2782514 (2202683-3362346) | 0.52 | 282795 |
| Isoleucine | 841158136 (804029451-8782868) | 848016440 (813093016-8829399) | 0.79 | 6858303 |
| Mannose | 14415390 (13424036-1540674) | 13730336 (12797864-1466281) | 0.33 | 685053 |
| Met-laurate | 50994558 (43786727-5820239) | 47469200 (40689478-5424892) | 0.49 | 3525358 |
| Glucose | 988189203 (948227835-1028151) | 954392653 (916804793-9919805) | 0.23 | 33796550 |
| Acetoacetate | 1271411 (975333-1567489) | 1223916 (945424-1502409) | 0.82 | 47494 |
| Met-caprate | 10005996 (8442618-1156937) | 11045103 (9574582-1251562) | 0.35 | 1039106 |
| Transaminase ratio (ASAT/ALAT) | 1.05 (0.91-1.19) | 0.95 (0.82-1.09) | 0.33 | 0.1 |
| Glucose clamp ratio (clamp girbw-60)/ clamp avg insulin) | 0.02 (0.02-0.02) | 0.02 (0.02-2.00) | 0.99 | 0.000016 |
| Valine | 170234100 (156937800-183530400) | 170035100 (157528500-183539400) | 0.98 | 1990151 |
| Histidine | 43399370 (41861940 - 44936800) | 448279400 (431934300 - 464624600) | 0.22 | 1428573 |
| Imidazole propionate | 3499907 (2872163 -4127651) | 3900503 (3233120 - 4567887) | 0.40 | 40059 |
|  | | | | |

# Supplementary Figure 1. Correlations matrix for predictors included in the analyses
